# Alfa-Fetoprotein-Producing Female Primary Urethral Adenocarcinoma with Neuroendocrine Differentiation

**DOI:** 10.1155/2019/3454037

**Published:** 2019-06-09

**Authors:** Takao Natsuyama, Yozo Mitsui, Masato Uetani, Shigeyuki Ohta, Masafumi Inoue, Koichiro Akakura

**Affiliations:** ^1^Department of Urology, Chiba-Nishi General Hospital, 270-2251 Chiba, Japan; ^2^Department of Urology, Japan Community Health Care Organization, Tokyo Shinjuku Medical Center, Tokyo, Japan; ^3^Department of Urology, Toho University Faculty of Medicine, 143-8540 Tokyo, Japan; ^4^Department of Pathology, Japan Community Health Care Organization, Tokyo Shinjuku Medical Center, Tokyo, Japan

## Abstract

We report an extremely rare case of an alpha-fetoprotein- (AFP-) producing female primary urethral adenocarcinoma with neuroendocrine differentiation (NED). The patient was a 65-year-old woman with a 2-year history of urinary frequency and voiding difficulty. Enhanced computed tomography showed an approximately 3.0×5.0-cm mass around the proximal urethra and bladder neck. Of examined tumor markers, serum AFP was elevated (48.3 ng/mL), while others including carcinoembryonic antigen were within a normal range. Transurethral resection of the tumor led to a diagnosis of carcinosarcoma of the urethra, with a radical cystourethrectomy and ileal conduit formation subsequently performed. The pathological assessment was poorly differentiated adenocarcinoma in the urethra. Immunostaining showed tumor cells strongly positive for AFP. In addition, some cancer cells were positive for CD56, chromogranin A, and synaptophysin, indicating focal NED. The tumor was finally diagnosed as an AFP-producing urethral adenocarcinoma with NED. Serum AFP was immediately normalized after surgery and no sign of tumor recurrence has been noted 2 years postoperatively.

## 1. Introduction

Tumor markers can provide valuable information regarding specific features as well as tumor burden and thus contribute to accurate disease diagnosis and prediction of therapeutic response. Elevated levels of carcinoembryonic antigen (CEA) and carbohydrate antigen (CA) 19-9 in serum are sometimes found in patients with urethral carcinoma [1]. However, elevation of serum alpha-fetoprotein (AFP) is extremely rare in association with this disease [2]. Furthermore, neuroendocrine differentiation (NED) is occasionally observed in various types of malignancy, including that of the genitourinary tract such as prostate cancer during an advanced disease stage [3], whereas the phenomenon is uncommon in cases of urethral cancer. Here, we present an extremely rare case of AFP-producing female primary urethral adenocarcinoma with NED.

## 2. Case Presentation

**Figure 1:**
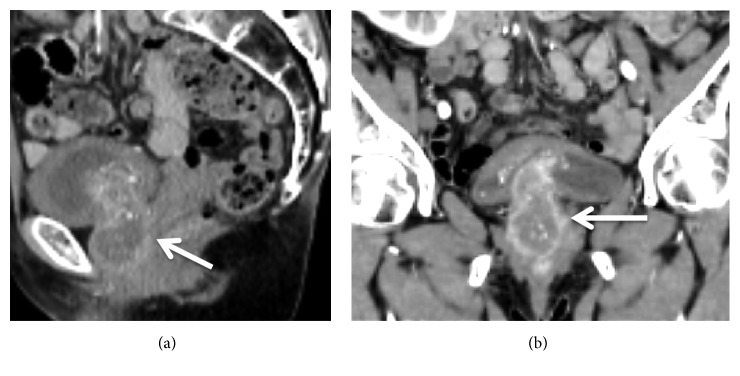
Abdominal enhanced computed tomography images showing a 3.0×5.0 cm mass with a heterogeneous contrasting effect between the urethra and bladder neck. (a) Sagittal section (arrow indicates tumor). (b) Coronal section (arrow indicates tumor).

A 65-year-old Japanese woman came to our hospital because of increased urinary frequency and dysuria over a 2-year period. Abdominal ultrasonography and cystoscopy findings revealed a broad based non-papillary tumor with a diameter of approximately 3.0 cm around the neck of the urinary bladder, with urine cytology findings positive for malignancy. Enhance computed tomography revealed a 3.0×5.0-cm mass with a heterogeneous contrasting effect between the urethra and bladder neck (Figure 1), though no distant metastasis was found. Additional examinations showed a serum AFP level of 48.3 ng/mL (normal <10.0 ng/mL), while CEA, CA19-9, and prostate specific antigen (PSA) levels were within normal ranges. Transurethral resection of the tumor between the urethra and bladder neck did not lead to a definitive diagnosis, but the possibility of carcinosarcoma of the urethra was indicated. Based on these findings, we diagnosed urethral cancer, clinical stage T3N0M0, and performed a radical cystourethrectomy procedure along with ileal conduit formation. Lymphadenectomy was not performed in this case.

**Figure 2:**
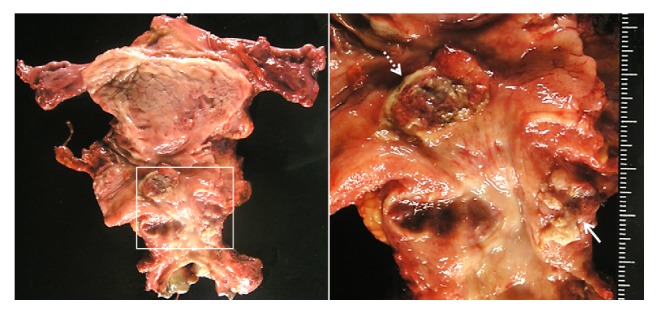
Macroscopic view of excised specimen. On the left is the full specimen and on the right is an enlarged image showing the tumor. The excised specimen had two ulcerated cancer lesions, one (3.0×1.8 mm in diameter, dotted arrow) around the trigon of the urinary bladder and the other (2.5×3.0 mm in diameter, arrow) on the proximal urethra.

**Figure 3:**
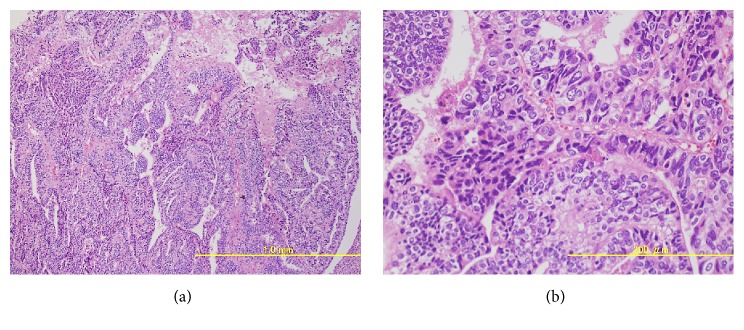
Histopathological findings. The diagnosis was urethral adenocarcinoma with various characteristics. (a) Hematoxylin and eosin stain, ×40. (b) Hematoxylin and eosin stain, ×200.

**Figure 4:**
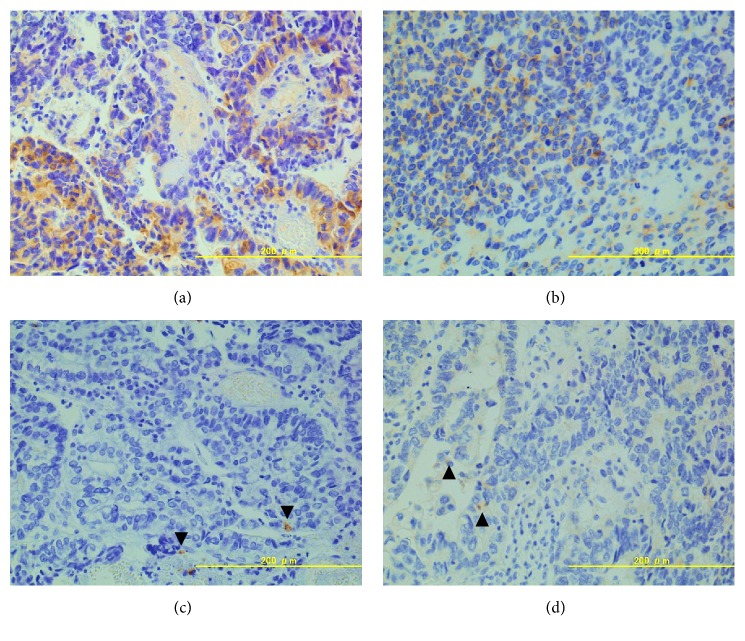
Immunohistochemical findings. (a) Tumor cells strongly positive stained for AFP (×200). (b) Positive staining for CD56 (×200). (c, d) Several cells were immunohistochemically positive for (c) chromogranin A (×200, arrowheads) and (d) synaptophysin (×200, arrowheads).

The total operation time was 8 hours 28 minutes and blood loss was 1081 mL. As shown in Figure 2, the excised specimen contained two ulcerated cancer lesions, one around the trigon of the urinary bladder and the other on the proximal urethra. Pathological assessment of the resected specimen revealed a poorly differentiated adenocarcinoma with various characteristics, such as clear cytoplasm and an NE tumor appearance (Figure 3). Immunohistochemistry was performed, which showed cells stained positive for AFP (Figure 4(a)), and negative for CEA, CA19-9, PSA, and p63. In addition, some cancer cells were positive for the NE markers CD56 (Figure 4(b)), chromogranin A (Figure 4(c)), and synaptophysin (Figure 4(d)). As these characteristics of the tumors were common to the two lesions, we considered that they were originally continuous lesion before transurethral resection. Finally, the morphologic and immunohistochemical findings confirmed a diagnosis of AFP-producing primary urethral adenocarcinoma with focal NED (the final tumor stage was pT3).

There were no peri-operative complications and the patient was discharged 37 days after surgery without adjuvant treatment. Serum AFP was immediately normalized after surgery and no signs of tumor recurrence including re-elevation of serum AFP have been noted 2 years postoperatively.

## 3. Discussion

We describe here the first known case of AFP-producing female primary urethral adenocarcinoma with neuroendocrine components. To the best of our knowledge, only a single case of urethral cancer with elevated AFP in serum has been reported thus far [2], while the phenomenon of NED has scarcely been reported to occur in patients with a primary urethral carcinoma. Thus, the present case is extremely unusual, as the patient showed both rare features concurrently.

Primary urethral carcinoma is a rare disease that accounts for less than 1% of cases of malignancy, with 5-year survival rates ranging from 40% to 60% [4, 5]. The ratio of female to male has been reported to be 2:3 and the most common histologic subtype among women is adenocarcinoma [6]. An adenocarcinoma can occur in a variety of tissues and organs, including the lungs, colorectum, and prostate, as well as others, and may occasionally be accompanied by NED [3, 7–9]. Interestingly, it has been reported that this phenomenon occurs only rarely, even in female patients with an adenocarcinoma in the urethral diverticulum [10]. Some adenocarcinomas with a diverticular form can arise from the proximal portion of the female paraurethral duct or gland, each of which expresses numerous endocrine cells [11, 12]; thus it is not surprising that cancer arising from these ducts exhibit focal NED. Furthermore, the proximal portion of the female paraurethral duct sometimes shows positive staining for PSA because it is embryologically homologous to the male prostate gland [12]. However, the existence of distinct subsets of paraurethral ducts showing negative staining for PSA is assumed. On the basis of such reported findings, Kato et al. presented diagnostic criteria, in which the phenomenon of NED is considered to indicate a female urethral adenocarcinoma with a diverticular form originating from the proximal portion, while positive for PSA staining is excluded from those criteria [11]. Although the present case was a primary urethral adenocarcinoma that did not contain diverticular components, focal NED was observed in cancer tissues with negative staining for PSA. A previous study suggested that a female primary urethral adenocarcinoma can arise from more than one tissue of origin [13]. Therefore, we considered that the cancer cells in our case may have been, at least in part, derived from the proximal portion of the paraurethral duct.

AFP is a protein normally produced by the liver and yolk sac of a developing fetus, and widely used as a serum biomarker for hepatocellular carcinomas and yolk sac tumors. In addition to those tumors, several types of malignancy, including lung, ovarian, and gastrointestinal cancer, are known to occasionally produce AFP [7–9], though an elevated level in serum is extremely rare in cases of urethral cancer. To date, the mechanisms underlying AFP production by these tumor cells has not been well elucidated. Interestingly, cancer with NED has been reported to be occasionally accompanied by an elevated serum AFP level, which is thought to be a marker of tumor cell dedifferentiation [7, 9, 14]. In addition, it is important to note that the urethra is embryologically derived from a cloaca containing AFP. On the basis of these reports, we speculated that some of the tumor cells in the present case had dedifferentiated and acquired an embryonic-like stemness, thus were subsequently capable of producing AFP.

When the serum level of AFP is found to be elevated, it is important to exclude the possibility of carcinoma metastasis to the liver or non-neoplastic liver injury. In the present case, laboratory examination results showed normal liver function, and CT imaging revealed no definite liver findings of metastasis or hepatobiliary lesions. In addition, immunohistochemical analysis showed that tumor cells were positive for AFP and the serum AFP level quickly returned to a normal range after surgery. Together, those results strongly suggested that AFP in our patient was produced by urethral cancer cells and the level in serum was correlated with tumor burden. In general, female urethral carcinoma is diagnosed as an aggressive disease in an advanced stage [6]. In addition, both NED phenomenon and AFP secretion are features indicating an aggressive malignant potential, as well as high propensity for metastasis to other organs [3, 7–10]. Thus, cancer patients with theses features require careful follow-up. We believe that monitoring the serum level of AFP can contribute to early detection of tumor recurrence and metastasis in such cases.
